# Loading mechanisms of ring helicases at replication origins

**DOI:** 10.1111/j.1365-2958.2012.08012.x

**Published:** 2012-03-15

**Authors:** Panos Soultanas

**Affiliations:** School of Chemistry, Centre for Biomolecular Sciences, University of Nottingham, University ParkNottingham NG7 2RD, UK

## Abstract

Threading of DNA through the central channel of a replicative ring helicase is known as helicase loading, and is a pivotal event during replication initiation at replication origins. Once loaded, the helicase recruits the primase through a direct protein–protein interaction to complete the initial ‘priming step’ of DNA replication. Subsequent assembly of the polymerases and processivity factors completes the structure of the replisome. Two replisomes are assembled, one on each strand, and move in opposite directions to replicate the parental DNA during the ‘elongation step’ of DNA replication. Replicative helicases are the motor engines of replisomes powered by the conversion of chemical energy to mechanical energy through ATP binding and hydrolysis. Bidirectional loading of two ring helicases at a replication origin is achieved by strictly regulated and intricately choreographed mechanisms, often through the action of replication initiation and helicase-loader proteins. Current structural and biochemical data reveal a wide range of different helicase-loading mechanisms. Here we review advances in this area and discuss their implications.

## Introduction

The ability to replicate is a universal characteristic of all living organisms. To produce descendants that are exact copies of the parental cell, the genetic information must be copied and transmitted from generation to generation. With the exception of a few viruses, the DNA double helix is the carrier of genetic information within the nucleotide sequences. Separation of the two strands by ubiquitous replicative DNA helicases reveals the parental nucleotide sequences for DNA polymerases to copy into new strands. Replicative helicases are closed ring structures with single DNA strands threaded through the ring during replication initiation via a process known as helicase loading. Although the problem of threading a very long linear DNA molecule through a circular helicase ring is fundamentally the same in all living organisms, a remarkable variety of loading mechanisms have evolved utilizing the basic principles of ring opening or ring assembly. Here we review recent advances in our understanding of helicase loading mechanisms in different organisms (Table 1). We discuss and compare mechanisms with direct or indirect involvement of specialized helicase-loader proteins, as well as self-loading mechanisms in the absence of helicase loaders.

**Table 1:** Ring opening or ring assembly mechanisms of helicase loading

Ring opening	Helicase loader	Ring assembly	Helicase loader
*E. coli* (DnaB)	+ (DnaC)	*B. subtilis* (DnaC)	+ (DnaI/DnaB)
*G. kaustophilus* (DnaC)	+ (DnaI)	SV40 (LTag)	−
Eukaryotes (MCM2–7)	+ (ORC/Cdc6)	Papilloma virus (E1)	−
*H. pylori* (DnaB)	−		
*P. aeruginosa* (DnaB)	−		
*P. putida* (DnaB)	−		
Bacteriophage T7 (gp4)	−		
TWINKLE	−		

## The bacterial DnaA replication initiator

In many organisms, DNA replication begins at specific chromosomal origins (*oriC*) where specialized replication initiator proteins bind to cause local melting of the DNA double helix and enable the loading of ring helicases. Replication initiators from all domains of life belong to a group of proteins known as AAA+ (ATPases Associated with various cellular Activities) proteins (for reviews see Iyer *et al*., 2004; Erzberger and Berger, 2006; Duderstadt and Berger, 2008; Wigley, 2009; Kawakami and Katayama, 2010). Their multimerization, DNA binding, duplex melting and helicase loading activities are co-ordinated by ATP binding and hydrolysis.

**Fig. 1:**
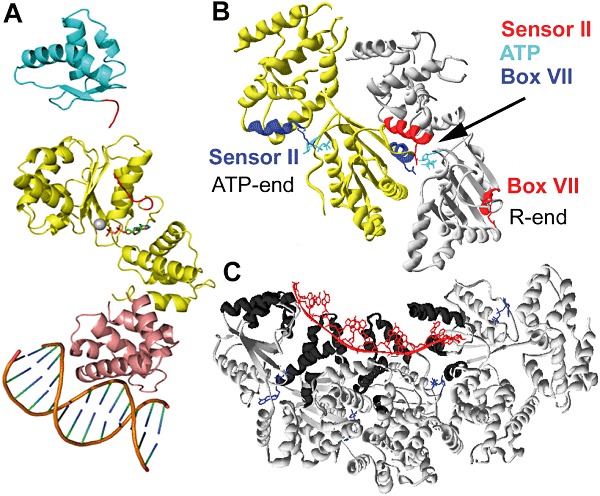
A. The domain organization of DnaA. Structures of the N-terminal helicase-interacting domain (domain I) are shown in cyan, domain III comprising the AAA+ subdomain IIIA that binds ATP and the lid subdomain IIIB in yellow, and the C-terminal domain IV that interacts with double-stranded DNA in pink. The flexible linker domain II is missing while the ATP and Mg^2+^ (grey ball) are shown in the active site of the AAA+ domain III. B. DnaA oligomerization. Two monomers of domain III are shown in yellow and grey with their arginines from Box VII and sensor II coloured in blue (yellow subunit) and red (grey subunit). The sensor II (red) of the grey monomer interacts *in cis* with the ATP (cyan) bound in its active site whereas the Box VII (blue) of the yellow monomer interacts *in trans* with the same ATP, as shown by the arrow. The ATP bound in the active site of the yellow monomer is shown with the relevant sensor II arginine defining the ATP-end of a filament, while the position of the Box VII of the grey monomer defines the R-end of the filament. C. The DnaA-ssDNA filament. A mini filament comprising four DnaA subunits bound to single-stranded DNA (red) and ATP (blue). The contiguous network of the α3/α4 and α5/α6 helices (domains III + IV) forming the single-stranded DNA binding surface lining the inside of the filament is highlighted in black (Duderstadt *et al*., 2011).

The replication initiator protein DnaA is strictly conserved across all bacteria. It comprises a helicase-interacting domain I linked to an AAA+ domain III via a linker domain II of variable length, and a double-stranded DNA interacting domain IV (Fig. 1A; reviewed in Leonard and Grimwade, 2011). The AAA+ domain III comprises two subdomains IIIA and IIIB, the latter known as the lid, with the ATP binding site situated at their interface. A helix, known as sensor II, from the lid contributes an arginine that interacts with the γ-phosphate of the bound ATP in the active site and keeps it in an open state, allowing a second arginine from another helix, known as Box VII, from the subdomain IIIA of a second DnaA molecule to dock *in trans* into the open active site through an interaction with the γ-phosphate of the bound ATP to form a dimer (Fig. 1B and Erzberger *et al*., 2006). The same structural arrangement in the ATP binding site of the second DnaA molecule allows docking of the arginine from the Box VII of a third DnaA molecule to form a trimer and so on. In this manner several DnaA molecules can interact to form a DnaA multimer. A helical insertion in their AAA+ domains (Iyer *et al*., 2004) protrudes away from the core structure and forces the interacting DnaA monomers to adopt a right-handed helical filament rather than a flat closed ring (Erzberger *et al*., 2006). Indeed, evidence of DnaA filament formation *in vivo* was recently reported in *Bacillus subtilis* (Scholefield *et al*., 2012). When the active site is empty, or when ADP is bound in the active site, the γ-phosphate–sensor II interaction is absent and the lid repositions itself changing the active site to a closed state. The Box VII arginine of a second DnaA molecule is then sterically blocked from docking into the closed active site preventing multimerization and filament formation. Mutating the Box VII arginine (R285 in *Escherichia coli*) prevents the formation of an active nucleoprotein complex, suggesting its importance in DnaA filament formation (Kawakami *et al*., 2005). In contrast, mutating the sensor II arginine (R334 in *E. coli*) reduces the ATPase activity but does not prevent DnaA-ATP oligomerization (Su'etsugu *et al*., 2001), suggesting that filament formation is not strictly dependent on the interaction of the sensor II arginine with the γ-phosphate of the bound ATP. Analogous mutations of the sensor II arginine in *Staphylococcus aureus* (Kurokawa *et al*., 2009), in *Caulobacter crescentus* (Fernandez-Fernandez *et al*., 2011) and in *E. coli* (Nishida *et al*., 2002) confirmed an ATPase defect and revealed an over-initiation phenotype. This is consistent with blocking ATP hydrolysis and stabilizing the DnaA filament leading to over-initiation. Therefore, binding and hydrolysis of ATP mediate a molecular switch mechanism (open versus closed active site) that regulates DnaA filament formation.

Modelling the binding of double-stranded DNA to the DnaA right-handed filament, using the crystal structure of domain IV bound to a double-stranded DnaA box (Fujikawa *et al*., 2003), reveals that the DNA wraps around the outside of the filament forcing positive supercoiling of the double helix (Erzberger *et al*., 2006). Compensatory negative supercoiling is then induced ahead of the DnaA–DNA filament which is thought to destabilize the double helix and enhance melting of an AT-rich region, known as the DUE (DNA Unwinding Element), within the *oriC*. Recent structural data revealed that the DnaA filament can also wrap around single-stranded DNA using a continuous network of alternating α3/α4 and α5/α6 helices within the central cavity of the filament (Fig. 1C and Duderstadt *et al*., 2011). The single-stranded DNA is stretched from 42 Å for a regular B-type helix to 65 Å which is proposed to actively enhance the melting of the duplex in the DUE.

## The bacterial helicase loaders

Some bacteria employ specialized helicase loaders that belong to the AAA+ family and assist helicase loading. *E. coli* and related organisms encode the helicase-loader DnaC, whereas *B. subtilis* and related organisms encode the helicase-loader DnaI (Koonin, 1992; Soultanas, 2002). DnaC and DnaI form tight complexes with their cognate or related ring helicases (Bárcena *et al*., 2001; Velten *et al*., 2003; Ioannou *et al*., 2006; Tsai *et al*., 2009) but they only share limited sequence homology, largely confined to their Walker A and B motifs characteristic of NTP-binding proteins (Soultanas, 2002). They comprise an AAA+ C-terminal domain and a smaller helicase-interacting N-terminal domain (Ioannou *et al*., 2006; Mott *et al*., 2008; Loscha *et al*., 2009; Tsai *et al*., 2009). The crystal structure of the AAA+ C-terminal domain of *Aquifex aeolicus* DnaC revealed a fold highly homologous to the DnaA AAA+ domain III (Mott *et al*., 2008). Despite the limited sequence homology between DnaC and DnaA, DnaC proteins have retained the Box VII and sensor II arginine fingers found in DnaA. In a manner similar to DnaA, binding and hydrolysis of ATP acts as a molecular switch to control multimerization into a spiral mini-helical structure similar to those formed by ATP-bound DnaA. This spiral structure is thought to be one way of opening the helicase ring, by sitting on one face of the flat helicase ring and forcing it to open.

Interestingly, the Gram-positive *Geobacillus kaustophilus* DnaI AAA+ C-terminal domain does not form such spiral helical structure (Tsai *et al*., 2009), while the *B. subtilis* DnaI N-terminal domain binds to the C-terminal tier of the ring helicase and has a novel fold held together by a structurally important Zn^2+^ ion (Ioannou *et al*., 2006; Loscha *et al*., 2009). The *B. subtilis* helicase-loader DnaI cooperates with a co-loader protein DnaB (not to be confused with the *E. coli* DnaB helicase) to load the replicative helicase DnaC (not to be confused with the *E. coli* helicase-loader DnaC) in a two-protein strategy (Velten *et al*., 2003).

The DnaB co-loader is found exclusively in low G+C content Gram-positive firmicutes together with another replication initiation protein, DnaD. *B. subtilis* DnaB is a structural homologue of DnaD comprising one DDBH1 and two DDBH2 (DnaD DnaB Homology 1 and 2) domains (Nunez-Ramirez *et al*., 2007; Marston *et al*., 2010). It is found in the cytosol and the bacterial membrane (Grainger *et al*., 2010), and is associated with the *oriC* and sites of replication–transcription conflicts where re-loading of the helicase and re-establishment of the replication fork is required (Merrikh *et al*., 2011; Soultanas, 2011). Atomic force microscopy and electron microscopy imaging of *B. subtilis* DnaB revealed a tetrameric structure forming a two-tier ring (Zhang *et al*., 2005; Nunez-Ramirez *et al*., 2007). One tier forms a closed ring and the second tier forms an open ring in a structure reminiscent of the *E. coli*γ clamp-loader complex (Nunez-Ramirez *et al*., 2007). The closed tier likely comprises the N-terminal DDBH1 and central DDBH2 domains that oligomerize and the open tier comprises the C-terminal DDBH2 domains which are sensitive to *in vivo* proteolysis that may be part of a regulatory mechanism preventing localization at the *oriC* (Grainger *et al*., 2010).

## Hexameric helicase ring loading by helicase loaders

### A. aeolicus

**Fig. 2:**
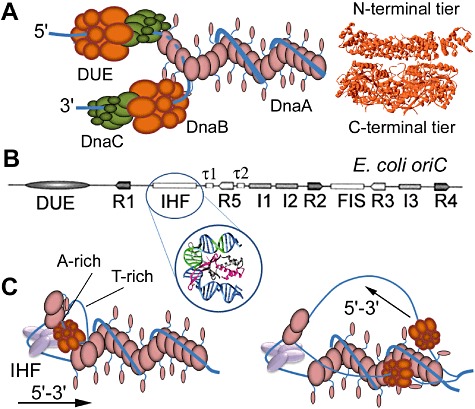
A. Helicase loader-mediated loading in *A. aeolicus*. A schematic diagram showing directional helicase loader-mediated and DnaA-mediated loading mechanisms on the 5′ and 3′ strands, respectively, based upon the *A. aeolicus* system (Duderstadt *et al*., 2011). The DnaA filament is shown to wrap double-stranded DNA around the outside with flexible helicase-interacting domains (domain I) projecting out from the filament. The filament extends into the 5′ strand of the melted DUE but in this case with the ssDNA in the interior of the filament. The helicase loader (green) forms a continuous heterofilament with the ATP-end of the DnaA-ssDNA filament, by docking the arginine from its Box VII into the ATP binding site of the DnaA, and delivers the helicase (brown) in the correct orientation onto the 5′ strand. The interactions of the flexible N-terminal DnaA domains with the helicase deliver it onto the 3′ strand in the opposite direction. A side-view of the *Bacillus stearothermophilus* DnaB helicase is shown with the characteristic two-tier (N-terminal and C-terminal tiers) ring structure of bacterial helicases (Bailey *et al*., 2007). B. Organization of the *E. coli oriC*. The relative positions of DnaA binding sites (R, I and τ sites), NAP (Nucleoid Associated Proteins) binding sites for IHF and FIS and the DUE are indicated. Binding of IHF between the R1 and R5 sites sharply bends the DNA, as indicated in the inset showing the crystal structure of IHF binding and bending double-stranded DNA. C. DnaA-mediated helicase loading in *E. coli*. A schematic model showing how two ring helicases are directionally loaded onto the *E. coli oriC* (Ozaki and Katayama, 2012). The DUE is cooperatively melted via binding of DnaA and IHF and the first helicase is loaded onto the bottom (A-rich) strand directionally. Subsequent translocation forward (in the 5′–3′ direction) melts a larger segment of the duplex allowing loading of the second helicase in the top (T-rich) strand and in the opposite direction. In both cases loading is mediated by the flexible N-terminal helicase-interacting domains of DnaA projecting out of the filament. For the sake of simplicity no helicase loader is depicted but it may participate indirectly in the process by binding onto the C-terminal tier of the helicase ring forcing opening of the ring for loading to proceed.

In *A. aeolicus*, the DnaC helicase loader cooperates with the replication initiator DnaA to load two ring helicases at the replication origin. The DnaA and DnaC nucleoprotein filaments described above are directional. One end of the filament terminates with a protein molecule (DnaA or DnaC) presenting an ATP-bound active site in the open state, defined as the ATP-end, and the opposite end of the filament terminates with a protein molecule presenting the arginine finger of Box VII, defined as the R-end (Fig. 1B and Mott *et al*., 2008). Initial binding of DnaA molecules to DnaA boxes, via the C-terminal domain IV, at the *oriC* forms a directional right-handed helical filament with the DNA double helix wrapped around the outside of the filament and the ATP-end directed towards the DUE. The constrained positive supercoiling induces compensatory negative supercoiling and weakening of the double helix within the DUE. Partial melting of the DUE at the ATP-end of the filament exposes the two DNA strands and the DnaA filament invades one of the strands forming an extended filament, with the single-stranded DNA bound along the contiguous network of the α3/α4 and α5/α6 helices inside the DnaA filament (Fig. 2A and Duderstadt *et al*., 2011). Therefore, the role of DnaA is initially to destabilize and actively melt the DUE at the replication origin.

Loading of the ring helicase by the helicase-loader DnaC takes place directionally only on the 5′ strand that has been invaded by the DnaA filament (Fig. 2A). DnaC forms a six-member ‘helical mini-filament’ interacting with the C-terminal tier of the ring helicase. It is not clear whether the helicase ring is open or closed at this stage, but an attractive hypothesis is that the helicase ring is forced to open when in complex with the DnaC mini-filament. The R-end of the mini-filament docks via its conserved arginine of Box VII into the open active site of the last DnaA at the ATP-end of the DnaA filament, forming a continuous DnaB/DnaC–DnaA heterofilament. In this configuration, the ring helicase is loaded directionally onto the 5′ strand (Fig. 2A). Loading of the second ring helicase onto the opposite anti-parallel 3′ strand takes place via an interaction of the DnaA with the helicase (Fig. 2A). It is not clear whether the helicase that is loaded onto the 3′ strand is in complex with DnaC or not. Either way, the free ring helicase or the helicase–DnaC complex could be loaded by DnaA. The flexible N-terminal domain I of DnaA interacts with the N-terminal tier of the ring helicase and should not sterically prevent the C-terminal tier from interacting with DnaC in a ternary DnaC–helicase–DnaA complex (Fig. 2A). If the helicase ring is open when in complex with DnaC, then it would be attractive to speculate that DnaA loads a helicase complexed with DnaC, instead of a free helicase. Alternatively, the interaction of the N-terminal domain I of DnaA with the N-terminal tier of the helicase ring could somehow force the ring to open through a conformational change during loading, without requiring the ring opening activity of DnaC. Conceivably, up to six DnaA molecules from the nucleoprotein filament could interact, via their flexible N-terminal domains I, with the six helicase molecules in the ring around the N-terminal tier which would strengthen an otherwise weak DnaA–helicase interaction.

### E. coli

The presence of an IHF (Integration Host Factor) binding site in the *E. coli oriC* interrupts the series of DnaA binding boxes and likely disrupts the formation of a contiguous DnaA filament within the *oriC* extending through into the DUE, as suggested in the *A. aeolicus* helicase loading mechanism (Fig. 2B). Hence, an alternative helicase-loading mechanism has been suggested which explains the important role of IHF and does not involve the direct interaction of DnaA with the helicase-loader DnaC (Ozaki and Katayama, 2012). The IHF binding site is located just before the high-affinity DnaA box R1 flanking the DUE (Fig. 2B). Binding of IHF at its site results in a sharp 160° DNA bend reversing the direction of the DNA helical axis within a very short distance (Rice *et al*., 1996 and Fig. 2B). The R1-DUE segment of the origin bends backwards and towards the DnaA filament, while binding of DnaA to the R1 and R5 regions is sufficient to melt the DUE in the presence of IHF (Ozaki and Katayama, 2012). As the direction of the DNA helical axis is reversed by IHF the DnaA filament extends into the T-rich single strand of DUE (Ozaki *et al*., 2006; Ozaki and Katayama, 2012). Loading of the first ring helicase is directed exclusively onto the opposite exposed A-rich strand. The first ring helicase is loaded directionally on the A-rich strand via a direct protein–protein interaction between the N-terminal domain of the DnaA bound to R1 and the N-terminal tier of the helicase ring (Fig. 2C and Ozaki and Katayama, 2012). The helicase then moves forward unwinding the duplex and exposing an extended segment of the opposite T-rich strand for a second ring helicase to be loaded in the opposite direction via protein–protein interactions between the flexible N-terminal domains of DnaA molecules in the extended filament (Fig. 2C and Ozaki and Katayama, 2012). The helicase-loading mechanism proposed in *E. coli* does not entirely exclude the mechanism proposed in *A. aeolicus*. However, the apparent lack of an interaction between the *E. coli* helicase-loader DnaC and DnaA (Schaeffer *et al*., 2005; Keyamura *et al*., 2009), compared with the two interacting orthologues in *A. aeolicus* (Mott *et al*., 2008), makes it unlikely that a DnaA–DnaC heterofilament forms in *E. coli* which is inconsistent with an *aeolicus*-type loading mechanism.

The *E. coli* loading mechanism does not envisage a direct role for the helicase loader and the question arises, what exactly is the role of the DnaC helicase loader during helicase loading? An important role of DnaC could be in ring opening, whereby a spiral DnaC binds and opens the helicase ring, preparing it to be loaded by DnaA onto an exposed single DNA strand (Ozaki and Katayama, 2012). This is a plausible mechanism as the DnaA–helicase and DnaC–helicase interactions are not mutually exclusive, as explained above. In this case the role of DnaC is indirect but essential. However, the prepriming complex in *E. coli* likely contains three DnaC monomers per DnaB hexamer (Makowska-Grzyska and Kaguni, 2010) and therefore, either the proposed DnaC spiral contains three monomers (compared with six monomers in the *A. aeolicus* DnaC spiral) or a spiral DnaC structure is not essential for DnaB ring opening.

### G. kaustophilus

**Fig. 3:**
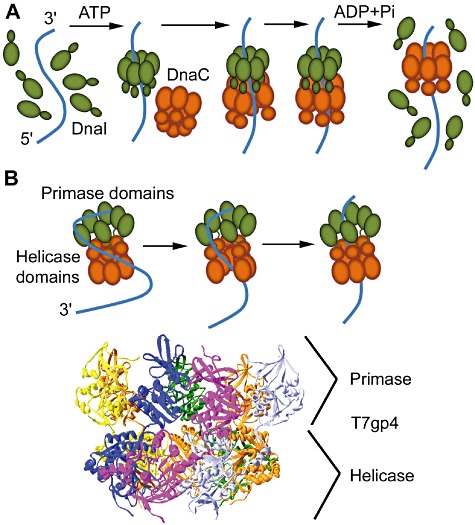
A. Schematic diagram showing DnaI-mediated loading of the *G. kaustophilus* ring helicase, DnaC. Monomers of DnaI (green) upon binding to ATP assemble into a hexameric ring around single-stranded DNA. The N-terminal tier of the DnaI ring interacts with the C-terminal tier of the helicase (brown) recruiting and opening up the helicase ring. Single-stranded DNA passes into the central channel of the ring. Binding of the DNA to the internal surface of the central channel stimulates the helicase activity and induces dissociation of DnaI (Tsai *et al*., 2009). B. Loading of helicase-primase bifunctional proteins. A helicase-primase protein initially interacts with the single-stranded DNA via its primase domains inducing an opening of the helicase ring with the DNA passing through the gap into the central channel. Binding of the DNA in the central cavity induces ring closure and activates the helicase-primase protein. The T7 gp4 primase-helicase crystallized as a heptamer (the seven subunits are coloured differently in the side view of the structure shown) but the translocating species along single-stranded DNA is believed to be the hexamer (Toth *et al*., 2003).

Interestingly, a ring opening role without the formation of a spiral structure has been proposed for the *G. kaustophilus* helicase-loader DnaI (Tsai *et al*., 2009). In this mechanism, DnaI monomers bind ATP and assemble directionally into a closed hexameric ring around single-stranded DNA with the N-terminal and C-terminal tiers towards the 5′- and 3′-ends of the DNA, respectively (Tsai *et al*., 2009 and Fig. 3A). Attempts to superimpose the *G. kaustophilus* DnaI AAA+ domain structure into the *A. aeolicus* DnaC helical filament by molecular modelling were unsuccessful due to steric collisions between the interfaces arising from additional N-terminal residues that are missing from the *aeolicus* DnaC (Tsai *et al*., 2009). Recruitment of the ring helicase in *G. kaustophilus* is mediated by direct protein–protein interactions between the C-terminal tier of the ring helicase and the N-terminal tier of DnaI ring, bringing the helicase in the vicinity of the single-stranded DNA and causing helicase ring opening and threading of the DNA directionally into the helicase ring cavity (Tsai *et al*., 2009 and Fig. 3A). It is not clear how a non-spiral helicase loader would achieve helicase ring opening but some yet unidentified local conformational change may still induce ring opening. The interaction of the DNA with the internal cavity of the helicase ring will activate ATP binding/hydrolysis, ring closure, helicase translocation forward in the 5′–3′ direction and DnaI disassembly (Tsai *et al*., 2009 and Fig. 3A). Alternatively, the recruitment of the primase DnaG may stimulate helicase activity and release from the helicase loader as has been suggested in *E. coli* (Makowska-Grzyska and Kaguni, 2010).

### B. subtilis

In *B. subtilis* the helicase-loading mechanism appears to be drastically different, as preformed *B. subtilis* ring helicase is inactive even in the presence of its pair of loaders, DnaI and DnaB (Velten *et al*., 2003). This observation argues against a ring opening mechanism in this system and supports instead a ring making loading mechanism. In this mechanism, the DnaI and DnaB work together to assemble from monomers a functional helicase hexameric ring directly onto single-stranded DNA (Velten *et al*., 2003). Presumably the *oriC* will be melted by the concerted action of DnaA and DnaD whose DNA stretching and untwisting activities (Zhang *et al*., 2006; 2008) could contribute towards DUE melting but the precise details of such a mechanism are still not known.

## Helicase loading in the absence of specialized loaders

### Primase-helicase bifunctional proteins (TWINKLE and T7gp4 DNA primase-helicases)

Some ring helicases utilize accessory domains to assist self-loading onto DNA. For example, linking primase proteins to a helicase is an efficient strategy for loading a helicase while at the same time maintaining close spatial proximity of two closely cooperating activities during DNA replication. Indeed, the structural homology of the C-terminal helicase-interacting domain of the bacterial primase to the N-terminal domain of the replicative ring helicase (Oakley *et al*., 2005; Syson *et al*., 2005) suggests a bifunctional self-loading primase-helicase bacterial ancestral protein which subsequently separated to individual primase and helicase proteins (Soultanas, 2005). The human mitochondrial helicase TWINKLE adopts a hexameric ring structure that can load onto closed circular DNA substrates without the assistance of a helicase loader (Jemt *et al*., 2011). The loading mechanism likely involves the primase-like N-terminal domain which binds single-stranded DNA (Farge *et al*., 2007) and acts as a helicase loader in a manner analogous to the ring opening mechanism mediated by the primase domain of the bacteriophage T7 gp4 helicase-primase protein (Ahnert *et al*., 2000; Toth *et al*., 2003). Binding of the TWINKLE primase domain to an initiation site induces a conformational change resulting in ring opening. Migration of the single-stranded DNA through the gap places it into the central cavity of the ring, followed by ring closure to complete the loading process (Fig. 3B). A helix-loop region that forms the linker between the primase and helicase domains of T7 gp4 appears to be important in ring opening, during helicase loading, as mutations of an alanine residue (A257) in this region result in defective loading (Patel *et al*., 2011) and non-viable phage (Lee and Richardson, 2004).

## Helicase domains fused to specialized *ori*-binding domains

### SV40 LTag

**Fig. 4:**
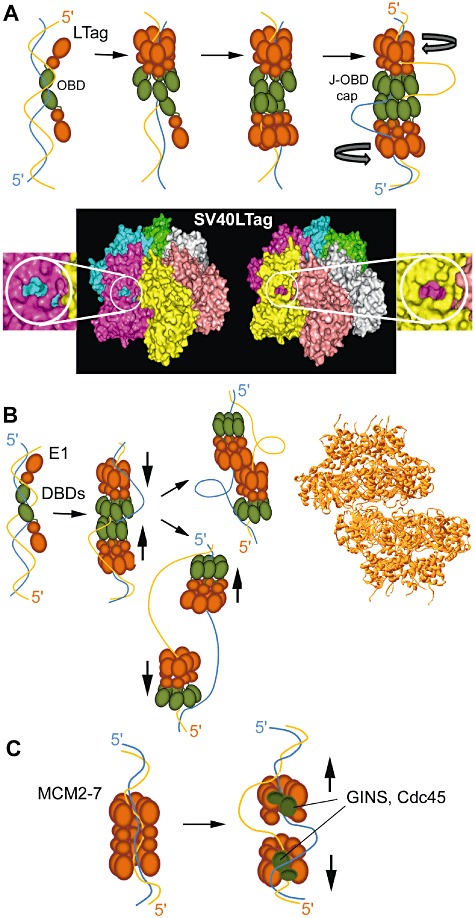
A. Self-loading of the SV40 LTag DNA helicase. Two monomers of LTag bind head to head to the SV40 core *ori* sequence via their OBDs (green). One hexamer assembles around the double-stranded DNA followed by the second hexamer in a cooperative manner. The J-OBD cap is sandwiched between the helicase rings and forms a network of interactions holding the dodecamer together. The double-stranded DNA is melted by the reverse ‘iris pumping’ action of the two helicases and the single strands loop out of the dodecamer from side channels at the periphery of the helicase rings. Six positively charged side channels that could potentially provide outlets for the unwound single strands can be seen in the crystal structure of the SV40 LTag helicase domains (Li *et al*., 2003) and two of them, in the pink and yellow monomers, are highlighted in two different side views (60° rotated around the vertical axis relative to each other) of the hexameric ring. B. Self-loading mechanism of the papilloma virus E1 DNA helicase. Two E1-ATP subunits bind cooperatively to the specific *ori* sequence via their DBDs (green) to form two head-to-head mini-filaments. Upon ATP hydrolysis a double hexamer forms. Each hexamer encircles opposite single strands and the DBDs form a cap sandwiched between the two helicase hexamers. The two hexamers translocate 3′–5′ in the opposite directions along their respective strands, as shown by the opposing vertical arrows, effectively pumping the double-stranded DNA towards the centre of the complex. Two ‘pumping mechanisms’ are possible. The two hexameric rings dissociate and keep on moving in opposite directions expanding the ensuing ‘bubble’ of the separated DNA duplex. Alternatively, the two hexameric rings as they cross each other adhere to each other via interactions of their helicase domains resulting in a double hexamer that keeps on pumping the double-stranded DNA towards the centre of the complex yielding a ‘rabbit ear structure’. The latter is supported by the crystal structure of the E1 helicase domains showing two hexameric rings associated with each other but not collinear as each encircles a different single strand (Enemark and Joshua-Tor, 2006). C. Loading of the MCM2–7 eukaryotic DNA helicase. MCM2–7 is loaded onto double-stranded DNA as a dodecamer, by the ORC/Cdc6 complex. Double-stranded DNA passes to the interior of the MCM2–7 ring channel through an opening between the MCM2 and 5 subunits. Binding of the dodecamer to double-stranded DNA distorts the DNA and additional pumping of the DNA by the two hexamers towards the centre of the dodecamer causes melting of the duplex inside the central cavity. Binding of the GINS/Cdc45 complex (green) to the periphery of the rings stabilizes open active forms of the rings, excluding different single strands from the central channels thus conferring 3′–5′ directionality along anti-parallel strands. The two hexamers then dissociate from each other and translocate in opposite directions.

Some helicases utilize specialized *ori*-binding domains. For example, the LTag (Large Tantigen) SV40 (Simian Virus 40) DNA helicase has been suggested to function as a dodecamer (Li *et al*., 2003; Gai *et al*., 2004). The LTag has additional specialized flexible and disordered J and OBD (Origin-Binding Domain) domains, N-terminal relative to the helicase domain. OBDs bind to four pentanucleotide repeats within the SV40 core *ori* sequence (Arthur *et al*., 1988; Luo *et al*., 1996). Initially two monomers of LTag bind to two of these pentanucleotides in a head-to-head orientation to assemble cooperatively the first and then the second hexamer head-to-head (Valle *et al*., 2006). During the assembly process, the J and OBD domains form a protein–protein interaction network (the J-OBD cap) sandwiched between two head-to-head hexameric rings. Structural distortions imposed on the DNA double helix and a squeeze-pump ATP-driven action of the two head-to-head hexameric helicases pulling the DNA in opposite directions force melting of the double helix (reviewed in Gai *et al*., 2010). The single strands then each exit the central ring channel through side gaps to form the functional dodecamer (Fig. 4A; Valle *et al*., 2006). The central channel of the SV40 LTag hexameric ring is large enough to accommodate double-stranded DNA, while six positively charged side channels that could potentially form the single-stranded DNA outlets of the unwound DNA can be seen in the crystal structure of LTag (Fig. 4A and Li *et al*., 2003).

### Papilloma E1 helicase

The papilloma virus E1 helicase employs a similar mechanism, using a DBD (DNA-Binding Domain) at its N-terminus (Enemark *et al*., 2002). The DBD binds to the double-stranded viral *ori* and distorts it through two separate DNA-interacting elements, the DNA-Binding Loop (DBL) and the DNA-Binding Helix (DBH), that individually bind only one of the two strands. The DBL extensively interacts with the DNA while the DBH has more limited interactions with the DNA and dissociates during strand separation. The DBDs also combine with a DNA-binding activity residing on the helicase domains to melt the *ori* and load two head-to-head hexamers each encircling the opposite strand (Fig. 4B; Schuck and Stenlund, 2005; Enemark and Joshua-Tor, 2006).

## Loading a double ring hexamer (dodecamer)

### The eukaryotic MCM helicase

Ring helicases often form head-to-head double hexamers (dodecamers) and some reports suggest that the dodecamer is the functional oligomer. One such helicase is the eukaryotic MCM replicative helicase. Although the formation of a dodecamer during SV40 LTag and E1 loading is reminiscent of the ORC (Origin Recognition Complex) and Cdc6 directed loading of archaeal and eukaryotic MCM double hexamers, the underlying loading mechanism of MCM is drastically different (reviewed in Remus and Diffley, 2009; Gai *et al*., 2010). The main feature of SV40 LTag and E1 DNA helicase loading is that they both assemble from lower oligomers (dimers) into a final dodecamer via what is essentially a ring making mechanism. But MCM loading does not involve ring making, and instead appears to involve loading of a preformed dodecamer by passing DNA through a side gap in the periphery of the ring. The eukaryotic MCM2–7 dodecamer is inactive at the end of mitosis and in G1 phase until it is activated in the S phase by a series of activating proteins including the four subunit GINS complex and Cdc45 (reviewed in MacNeill, 2010; Bell, 2011). Recent single molecule data revealed that lambda DNA was replicated by a pair of diverging replisomes assembled from *Xenopus* egg extracts (Yardimci *et al*., 2010). This is incompatible with a dodecameric model where two head-to-head helicases drive two replication forks pumping the DNA single strands simultaneously in opposite directions. An alternative model, whereby the MCM2–7 dodecamer is loaded in a pre-assembled form onto double-stranded DNA through a gap between the MCM2 and 5 subunits (Bochman *et al*., 2008) followed by binding of GINS and Cdc45 at the periphery of the MCM ring sealing the side gap and trapping one of the single strands outside the ring, could explain how the two MCM2–7 ring helicases are activated (Fig. 4C). The two hexameric MCM2–7 rings then detach and translocate in opposite directions separating the DNA strands through a molecular plough mechanism just like the bacterial helicases (Botchan and Berger, 2010). The resulting diverging replication forks are driven independently from each other (Fig. 4C).

### *Helicobacter pylori* and *Pseudomonas*

Evidence that ring helicases may also be loaded onto DNA independently of helicase loaders was provided by genetic complementation studies using the *Helicobacter pylori* helicase HpDnaB. HpDnaB complements a conditionally lethal *E. coli dnaB* allele and two different *E. coli dnaCts* mutants when expressed *in trans* (Soni *et al*., 2005). These data suggest that HpDnaB can bypass the need for a viable helicase loader. *H. pylori* does not appear to code for a helicase-loader orthologue in its genome (Nitharwal *et al*., 2011). Furthermore, *Pseudomonas aeruginosa* and *Pseudomonas putida* replicative helicases can be loaded at the *oriV* replication origin of the broad-host-range RK2 plasmid by plasmid encoded replication initiation proteins in collaboration with the host replication initiation protein DnaA without requiring a *de novo* helicase loader (Caspi *et al*., 2001).

Passing of DNA through a side gap at the periphery of the ring may also be the mechanism of loading of the HpDnaB helicase. A model of the dodecameric HpDnaB by docking the crystal structure of the C-terminal domain into an electron density map obtained by electron microscopy revealed two head-to-head ring hexamers with relatively large peripheral side gaps between the individual subunits, similar to SV40 LTag and MCMs (Stelter *et al*., 2012). Since the *H. pylori* genome lacks an identifiable helicase loader, a loading mechanism broadly similar to that described for the archaeal and eukaryotic MCMs may be utilized to load the dodecamer, by threading the DNA strands through the side gaps into the interior channel of the ring. It is not clear at this stage whether HpDnaB is loaded as a dodecamer onto double-stranded DNA or onto single-stranded DNA produced by DnaA-mediated *oriC* melting. In the former case, the DNA double helix will have to melt inside the central ring channel and one of the strands will have to pass through the side channels back out from the channel, leaving only one DNA strand threaded through the ring before the onset of translocation. Opposite strands must be excluded for each of the two hexameric ring channels to ensure bidirectional loading, but it is not immediately obvious how this can be achieved. It is also not clear how HpDnaB could exclusively target the *oriC* instead of random single-stranded and/or double-stranded DNA. DnaA-mediated directional loading may target the HpDnaB to *oriC*, but a high-throughput yeast two-hybrid screen of the *H. pylori* proteome failed to detect a DnaA–HpDnaB interaction (Rain *et al*., 2001). A strong DNA-binding motif was recently identified in the C-terminal domain of HpDnaB. Structural modelling suggested that six of these motifs are situated symmetrically around the rim of the C-terminal tier and are masked by the N-terminal domains within the HpDnaB hexamer (Nitharwal *et al*., 2012). Repositioning of the N-terminal domains through protein–protein interactions could expose the strong DNA-binding motifs of the C-terminal tier, and could act as a regulatory mechanism to prevent indiscriminate binding of HpDnaB to any exposed single-stranded DNA. The same regulatory mechanism could play a role during loading of HpDnaB at *oriC* (Nitharwal *et al*., 2012). However, it is not clear whether these motifs are masked or exposed in the dodecameric HpDnaB structure.

## Conclusion

It is now becoming increasingly apparent that although the basic problem of loading a ring helicase onto a ‘linear’ DNA lattice is the same, biological systems have evolved different mechanistic solutions to solve the same fundamental problem. Such diverse solutions utilize the basic principles of ring opening or ring assembly and have likely evolved because of (i) different architectures of replication origins requiring either specialized sets of different loading proteins or no loading proteins at all, (ii) subtle differences in helicase structures and mechanisms often associated with additional activities such as primer synthesis during replication, (iii) strict regulatory requirements to comply with complex life cycles, or (iv) genome economy when additional genes coding for accessory loading proteins need to be eliminated.
